# Agenesis of Azygos Vein in a Case of Esophageal Atresia and Tracheoesophageal Fistula

**Published:** 2013-07-01

**Authors:** Bilal Mirza, Muhammad Saleem

**Affiliations:** Department of Pediatric Surgery, The Children’s Hospital and the Institute of Child Health Lahore, Pakistan

**Dear Sir**

A 2-day-old female neonate, weighing 2.7kg, presented with salivation, frothing, and choking at feeding attempts. There was a history of polyhydramnios in mother. The baby was a product of non-consanguineous marriage and delivered at term by normal vaginal delivery in a private hospital. General physical examination was unremarkable. There were no visible associated anomalies in the baby. Chest and upper abdominal radiograph showed feeding tube curled just beyond thoracic inlet and a big gastric shadow. Laboratory investigations were within normal limits. Patient underwent right thoracotomy which revealed agenesis of azygos vein (Fig. 1). Primary esophageal repair was done with mild anastomotic tension. Postoperative recovery was uneventful. Echocardiogram done postoperatively showed structurally and functionally normal heart. Patient was discharged in good general condition. 

**Figure:**
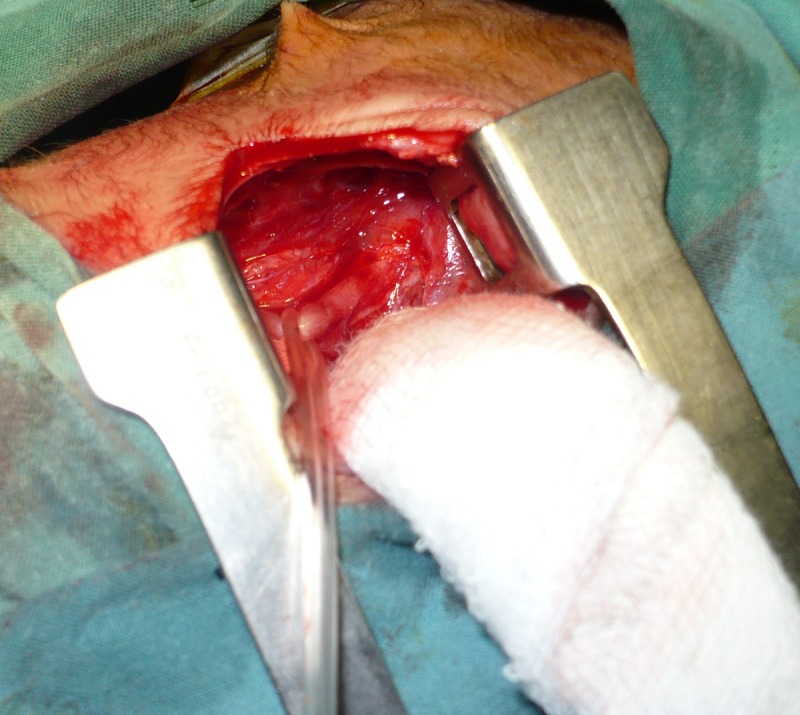
Figure 1: Absent azygos vein. Sling is encircling distal esophageal pouch showing communication with trachea.

Azygos vein commences as a continuation of right ascending lumbar vein and completes its course by draining into superior vena cava. It receives blood from intercostal veins, hemi-azygos and accessory azygos veins, pericardial veins, bronchial veins, and various esophageal veins etc. Azygos vein has been a subject of research in patients of esophageal atresia (EA) and tracheoesophageal fistula (TEF) to assess its prognostic value as regards to postoperative pneumonitis, anastomotic leakage, and ultimate outcome [1-3]. Its preservation in patients with EA/TEF abates postoperative complications. Patients of EA/TEF have a plethora of associated cardiac anomalies including septal defects, tetrology of fallot, patent ductus arteriosus, coarctation of aorta, left superior vena cava, and right sided aortic arch [4-7]; only few mentioned absence of azygos vein [4, 6]. Azygos vein helps identify level of TEF which mostly lies just below the crossing of azygos vein and vagus nerve. Its absence may result in inadvertent ligation of bronchus or aorta in beginner’s hands. Arbell et al [6] reported absence of azygos vein associated with left superior vena cava and advocated wide mediastinal dissection for identification of level of TEF. We, however, did not encounter any difficulty in identification of level of TEF. Following the vagus nerve was quite helpful in identification of lower esophageal pouch; moreover, aspiration of esophageal pouch with insulin syringe may help distinguish it from major vessels.

## Footnotes

**Source of Support:** Nil

**Conflict of Interest:** Authors belong to editorial board however they were not involved in evaluation and decision making about the manuscript.

## References

[1] Upadhyaya V D, Gangopadhyaya A N, Gopal S C, Upadhyaya A, Sharma S P, Gupta D K (2007). Is ligation of azygos vein necessary in primary repair of tracheoesophageal fistula with esophageal atresia?. Eur J Pediatr Surg.

[2] Sharma S, Sinha S K, Rawat J D, Wakhlu A, Tandon R K (2007). Azygos vein preservation in primary repair of esophageal atresia with tracheoesophageal fistula. Pediatr Surg Int.

[3] Rashid K A, Maletha M, Khan T R, Wakhlu A, Rawat J, Kureel S N (2012). Esophageal anastomosis medial to preserved azygos vein in esophageal atresia with tracheoesophageal fistula: restoration of normal mediastinal anatomy. J Neonat Surg.

[4] Gupta D K, Arora M, Srinivas M (2001). Azygos vein anomaly: the best predictor of a long gap in esophageal atresia and tracheoesophageal fistula. Pediatr Surg Int.

[5] Arslan G, Ozkaynak C, Cubuk M, Sindel T, Lüleci E (1999). Absence of the azygos vein associated with double superior vena cava--a case report. Angiology.

[6] Arbell D, Golender J, Khalaileh A, Gross E (2008). Search for the azygos: a lesson learnt from a case with left superior vena cava, esophageal atresia and tracheo-esophageal fistula. Pediatr Surg Int.

[7] Arsalan G, Cubuk M, Ozkaynak C (2005). Absence of the azygos vein associated with left superior vena cava. Euro J Radiol Extra.

